# 

**Published:** 1867-09

**Authors:** 


### Dental Register.

EDITED BY

J. TAFT & G. WATT.

SEPTEMBER, 1867.

MONTHLY:
THREE DOLLARS PER ANNUM, IN ADVANCE.

CINCINNATI:
WRIGHTSON & CO., Printers, 167 WALNUT STREET.

